# Common and Unique Barriers to the Exchange of Administrative Healthcare Data in Environmental Public Health Tracking Program

**DOI:** 10.3390/ijerph18084356

**Published:** 2021-04-20

**Authors:** Mikyong Shin, Charles Hawley, Heather Strosnider

**Affiliations:** 1 Environmental Public Health Tracking Section, Division of Environmental Health Science and Practice, National Center for Environmental Health, Centers for Disease Control and Prevention, Atlanta, GA 30341, USA; hstrosnider@cdc.gov; 2 National Association of Health Data Organizations, Provo, UT 84606, USA; chawley@nahdo.org

**Keywords:** tracking program, hospitalization, emergency visits data, NAHDO, data quality

## Abstract

CDC’s National Environmental Public Health Tracking Program (Tracking Program) receives administrative data annually from 25–30 states to track potential environmental exposures and to make data available for public access. In 2019, the CDC Tracking Program conducted a cross-sectional survey among principal investigators or program managers of the 26 funded programs to improve access to timely, accurate, and local data. All 26 funding recipients reported having access to hospital inpatient data, and most states (69.2%) regularly update data user agreements to receive the data. Among the respondents, 15 receive record-level data with protected health information (PHI) and seven receive record-level data without PHI. Regarding geospatial resolution, approximately 50.0% of recipients have access to the street address or census tract information, 34.6% have access to ZIP code, and 11.5% have other sub-county geographies (e.g., town). Only three states receive administrative data for their residents from all border states. The survey results will help the Tracking Program to identify knowledge gaps and perceived barriers to the use and accessibility of administrative data for the CDC Tracking Program. The information collected will inform the development of resources that can provide solutions for more efficient and timely data exchange.

## 1. Introduction

The Centers for Disease and Control and Prevention (CDC) National Environmental Public Health Tracking Program (Tracking Program) collects, analyzes, and distributes standardized data on many environmental health topics from a variety of national, state, and local partners [1,2]. The Tracking Program drives public health actions that protect people from harm resulting from exposure to environmental contaminants [3,4]. Nearly all states collect inpatient discharge and emergency department (ED) visit data from every licensed healthcare facility within their state. Inpatient and ED data are a major source of health outcomes data for the program’s National Environmental Public Health Tracking Network, a web-based system of environmental health data and information. The data from states are essential for filling the “environmental health data gap” to document potential links between environmental hazards and illness and disease [5].

The Tracking Program annually receives inpatient and ED data from 25–30 states. Health outcomes are reported for patients treated for asthma, chronic obstructive pulmonary disease, carbon monoxide poisoning, heart attack, and heat stress illness. Inpatient and ED data, along with some other types, such as data on ambulatory surgery visits, are often referred to collectively as administrative data [6], hospital data, or hospital discharge data, and are an important source of information on morbidities. The Tracking Program uses the data to inform policy and practice decisions and enhance the local analytical workforce and information infrastructure. Administrative data can also play a critical role in environmental public health surveillance, strategic planning, and public health response [7].

The National Association of Health Data Organizations (NAHDO) has been a Tracking Program partner since 2007. NAHDO has worked toward improving access to hospital data sources for measuring health outcomes [8]. The quality of hospital data affects the usefulness of the data [9,10]. Improving the timeliness and validity of existing hospital data sources is a mutual goal of the Tracking Program and its partners to increase the capacity to produce standardized and actionable data and measures [9,10,11,12].

The goal of this survey was to understand the knowledge gaps and perceived barriers to use and accessibility of hospital data for the CDC Tracking Program. The survey results will be used to evaluate the Tracking Program’s ongoing data call process and routine data validation and data sharing practices. The results will also help the Tracking Program improve standardized guidance and evaluation activities to support recipients in submitting data to the program.

## 2. Materials and Methods

### 2.1. Survey Design

The CDC tracking program conducted a cross-sectional survey among principal investigators or program managers of the 26 funded programs to improve access to timely, accurate, and local data. The tracking program and NAHDO collaboratively designed a survey instrument to assess common and unique barriers to the exchange and use of administrative data used by the Tracking Program. The 38 survey questionnaire was designed to assess six aspects of administrative data: (1) data source (2 questions), (2) data providers and user agreement (11 questions), (3) acquired data attributes (6 questions), (4) data from bordering states (7 questions), (5) data quality and validation (9 questions), and (6) partnership with data agency or organization (3 questions).

### 2.2. Data Collection

The draft survey questionnaire was pilot tested with three volunteer states (Connecticut, Massachusetts, and Michigan). A total 46 survey questionnaire was finalized based on feedback from those states on survey content, logic, clarity, structure, and response time. All feedbacks from the pilot test are shown in Supplement Table S1.

A pre-notice was sent by email to all 26 recipients one week before launch to announce the 2019 survey. Participating states included: Arizona, California, Colorado, Connecticut, Florida, Iowa, Kansas, Kentucky, Louisiana, Missouri, Maine, Maryland, Massachusetts, Michigan, Minnesota, New Hampshire, New Jersey, New Mexico, New York City, New York State, Oregon, Rhode Island, Utah, Vermont, Washington, and Wisconsin. The survey pre-notice asked that each recipient identify the principal investigator or program manager with the most knowledge about their administrative data. The pre-notice explained the purpose of the survey and how to respond.

Survey questions were emailed to respondents before launch so they could gather information. At launch a week later, a survey invitation was emailed to recipients. It included a hyperlink to an electronic version of the survey that respondents could use instead of the paper version, whichever the participants chose to complete. Nonresponsive recipients received a weekly reminder email each of the two weeks after launch. A survey closing note was sent to all recipients three weeks after launch.

### 2.3. Statistical Methods

Data were analyzed using descriptive statistics and summarized by the following data characteristics: type, provider, schedule, protected health information (PHI) inclusion, spatial resolution, elements to identify transfers, duplicates removal, how data problems are corrected, exclusions, and use for the Tracking Program. Data agreement types, time lag in the data, and information sharing with border states were also described.

## 3. Results

A total of 26 professionals from 26 tracking programs responded to the survey (100% response rate). Among those respondents, 16 (61.5%) had more than 4 years of professional experience with tracking programs, 5 (19.2%) had more than 13 years of experience, and 5 (19.2%) had less than 3 years of experience.

All 26 state programs (25 states and New York City) currently receive or access inpatient discharge data, but four states do not have access to ED visit data. In addition to the required inpatient or ED data, some states have access to observational stay data (eight states) or an all-payer claims database (six states). Eight states receive data from their hospital association; the other states receive data from their state health department, agency, commission, or board (Table 1). Among respondents, 15 (57.7%) received record-level data with protected health information, 9 (26.9%) received record-level data without PHI, and 2 (7.7%) received data with aggregated PHI. Survey results also showed that 8 (30.8%) respondents have access to street addresses, 3 (11.5%) have access to census tract information, and 14 (53.8%) have access to ZIP code or other sub-county geographies such as a town (Table 1). 

Transfer cases are identifiable by all but one state. Sixteen states (61.5%) have a combination of variables for identifying transfer cases, such as age, date of birth, and date of admission. Six states (23.1%) receive patient hospital identification (ID), and three states (11.5%) receive flags for transfer from data providers. Among respondents, 16 strongly emphasized the need for additional data elements (e.g., patient ID, full residential address, and geocoded data) to process data efficiently and identify transfer cases or sub-county geographies. Data providers (*n* = 12, 46.2%) or state programs (*n* = 9, 34.6%) remove duplicate data as a validation procedure. Almost one third of the programs (9, 33.3%) ask data providers to clean data by correcting errors and resubmitting after they validate the data (Table 1).

**Figure 1:**
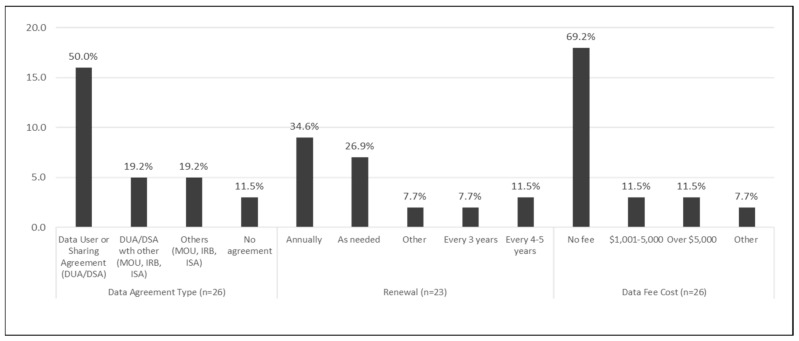
Data Sharing Agreement, 26 participating U.S. states *—CDC Public Health Tracking Program, 2019. * Twenty-five states and New York City.

Most states (88.5%) establish and maintain at least one data user agreement (DUA) or various types of combined agreements: 13 states only have a DUA or data sharing agreement (DSA) in place (50.0%), 5 states maintain a DUA with other combined agreements (e.g., memorandum of understanding, interdepartmental service agreement, or institutional review board exemption) (19.2%), 5 states have other types of agreements (19.2%), and 3 states do not have any agreement (11.5%) (Figure 1).

**Figure 2:**
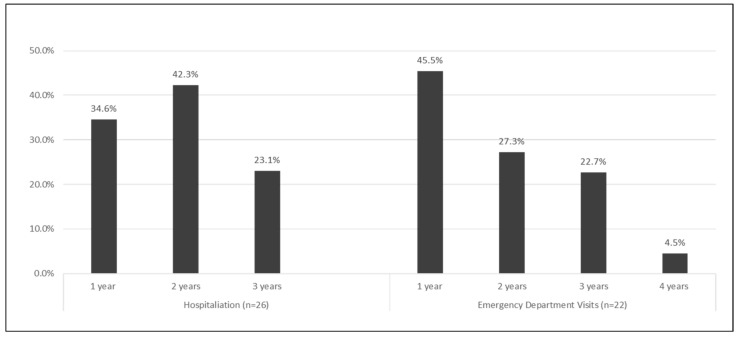
Data Lag Period, 26 participating U.S. states * —CDC Public Health Tracking Program, 2019. * Twenty-five states and New York City.

Most of the states receive data from their suppliers annually (61.5%) or quarterly (30.8%). Most states do not pay a fee to access the data, but six states pay more than $1000 for data access (Figure 1). The most recent period for which a program had received data ranged from 2015 to 2019. For the 2019 Tracking Program data call, 20 states were able to submit hospitalization data up to 2017 and emergency department (ED) visits data (76.9%, 72.8%) (Figure 2).

Only three states (11.5%) receive data from all their bordering states, and six states (23.1%) receive data from some bordering states (Table 2). Six states (Florida, Maine, Maryland, Massachusetts, New York State, and Oregon) attempted to obtain border data but still do not have border data. Data from bordering states typically lag 1–2 years behind in-state data collection. Seventeen recipients (65.4%) do not receive any hospital and ED data when their residents are admitted to hospitals or ED from bordering states. The survey found that some states pay to obtain data on residents discharged from facilities in bordering states. Nine states and one city (Arizona, California, Colorado, Connecticut, Iowa, Kentucky, Louisiana, New Jersey, New York City, Rhode Island, and Utah) never attempt to obtain border data.

## 4. Discussion

This survey revealed common and unique barriers for exchange of administrative data in the Environmental Public Health Tracking Program. Challenges to exchanging data with the tracking program that recipients encounter include (1) timeliness, (2) data granularity, (3) data acquisition from bordering states, and (4) data cleaning.

### 4.1. Timeliness

Along with comparability, completeness, and validity, timeliness is one of the most important data quality indicators [8,9]. Rapid reporting is a vital priority in cancer registry, injury prevention, birth defect registry, and immunization surveillance programs [11,12,13,14,15]. Real-time epidemic predictive modeling relies on timely data to forecast geographic disease spread and obtain case counts to inform better public health interventions, and is especially valuable during disasters and outbreaks [16,17].

The survey showed that challenges with receiving data from the provider, complex internal data processes, or incomplete data are responsible for data lag. To decrease lag time and variation from internal data processes across the state programs, a standardized DUA or DSA with a shared time frame is highly recommended, whether partnering intra-agency, inter-agency, or externally [18]. A national standard could include requirements for data layout and format, data quality benchmarks, a defined process for making corrections (especially for critical elements), and methods and timelines for exchanging data. State partners benefit from establishing working relationships and formal agreements with their hospital data suppliers. Data stewards might benefit from similar defined processes within their state data acquisition requirements and contracts.

Several studies have demonstrated the feasibility of chronic disease surveillance using data from electronic health records (EHR) [19,20]. EHR data tend to be more timely than data from traditional public health surveys or systems and can be available at a finer geographic level [20]. A recent study provided evidence of nationwide surveillance of asthma prevalence and asthma ED visits using EHR data from a multisite collaboration across the US [21,22,23]. Asthma prevalence estimates produced using EHR data were comparable to those produced using data from established national and state-level surveys [21]. Timely health outcome data can support responses to public health incidents such as wildfires, extreme heat, or other extreme weather events [23], and Tracking Program partners could explore obtaining EHR data in collaboration with their state recipients for more timely administrative data at a finer geographic level than county.

### 4.2. Data Granularity

State programs have attempted to receive data elements such as patient ID, Social Security Number, and full residential address or geocoded information. The survey showed that 16 (61.5%) of the state programs have successfully obtained the record-level identifiable data set with PHI or health outcomes at a finer geographic level (e.g., census tract, ZIP code). However, restrictions on the use of identifiable or sub-county level data vary by state, data source, or data supplier. Patient identifiers can be an important element for spotting duplicate records and out-of-state hospitalizations or transfer cases across hospitals or states.

A growing need to better understand the relationships between environment, behavior, and health has led to increased demand for small-area data [24,25]. Many state programs traditionally track aggregated hospital data by county, but recently are providing additional support for small-area data sharing [26]. Despite current limitations, small geographic area data that can be easily accessed and updated have become essential for targeting public health programs and services.

To access critical data elements, state programs need effective communication with their data suppliers (i.e., hospital associations and state agencies). A first step might be to determine whether the data steward has sufficiently granular data to support more detailed analysis. If so, programs could engage in process improvement through establishing or updating contractual requirements, preferably using a standardized data agreement. If granular data are not available, one course of action is partnering with the data steward to suggest and implement process improvement plans. A standard guidance and analyses plan for data granularity should be shared with tracking programs and partners to protect patient confidentiality and privacy, in accordance with relevant federal and state laws and regulations and department policies [24,27]. Data stewards and users should consider the challenges of obtaining health-related data in small geographic areas to balance the needs for data sharing and privacy [27,28].

### 4.3. Acquiring Data from Border States

States often have not received data for residents who were hospitalized or visited an ED located in the bordering states. Having timely and complete data for all residents is important to calculate annual trends. The survey showed that cross-border data exchange is complicated and varies by state, making it difficult to summarize (Table 2). Only three states exchanged data with bordering states, and 12 programs have not attempted to access data from bordering states. Complete data are important for environmental studies seeking to understand proximity to the health care services. In addition to the socio-economic status or patient-level factors such as race/ethnicity, the travel distance to the treating facility is likely to affect the course of diagnosis, subsequent treatment, and disease outcomes [29]. Acquiring all cross-jurisdictional data is essential to estimate health outcomes and to capture all exposures related to environmental hazards. 

The cross-border exchange of health data involves ethical, regulatory, and organizational issues converging on technical aspects such as interoperability and cybersecurity [30]. The State and Territorial Exchange of Vital Events (STEVE) system, for example, quickly provides vital records data to other jurisdictions and authorized public health and administrative programs, while also ensuring the security and privacy of the data during transmission [31]. Surveillance data exchange between the public health departments of the District of Columbia, Maryland, and Virginia reduced the number of cases misclassified as District of Columbia residents and reduced the number of cases with duplicates [32]. CDC’s tracking program and NAHDO can help state health departments communicate with each other and develop an internal data user agreement process to enable data exchange with bordering states. NAHDO also can assist with process development, process improvement, and collaboration among interested data stewards and hospital associations.

### 4.4. Data Cleaning

Data validation is critical for ensuring valid analytic results for any projects using health records and environmental monitored data [33,34]. Challenges can result from the initial delay from the responders, staff turnover, high caseloads, lack of resources, and competing prioritization in the health department [34]. It is critical to learn if data cleaning and duplicate resolution processes are inconsistent across the tracking state programs. Only 46.2% of state programs did data cleaning work before submitting data. The tracking program has provided tools, documents, and technical support for data cleaning. The program also encourages recipients to use generic SAS software scripts that Tracking program developed and a validation protocol for basic data cleaning and standardized data preparation.

After state programs submit their annual administrative data, the tracking program performs multiple layers of data validation to improve data quality. These data validation processes include a simple check for duplicates, descriptive statistics, and trend analysis that compare current data to archived data. In 2018, state programs resubmitted 1–2% of 217 total data feeds because the data failed validation checks.

As a part of the approach to data cleaning and routine analysis, the tracking program initiated a journal club to share and develop a framework for routine analysis, from data validation to analyses. State programs voluntarily present their work with data and receive feedback from the peer data scientists and a CDC senior statistician. Tracking program recipients and partners also participate in various hospitalization content workgroups to share data needs and expertise across the collaborative network.

The tracking program’s data provide valuable information for environmental health response, understanding disease related to environmental hazards, and taking actions to prevent or control diseases [4]. The program provides recommendations on best practices for data exchange and how to address barriers and gaps it identifies. However, states greatly vary in the effort they put into setting up new data user agreements or in amending established agreements. Coordinated efforts are needed to assess variation in data use agreements between states, and significant effort is needed to standardize such agreements.

Current data exchange methods prompt important questions about how to securely share more complete, timely, and accurate de-identified and linkable data with the tracking program. Tracking programs can gain a baseline understanding of state administrative data by accessing NAHDO’s state data agency profiles (an inventory of agency governance, laws, policies, and data scope) [8].

## 5. Conclusions

Results from this survey will help the tracking program understand perceived barriers to use and accessibility of administrative data for the CDC tracking program. The state profiles and information collected will inform the development of resources that can provide solutions for more efficient and timely data exchange. The collected information will be used to improve the ongoing data call process, including routine data validation and data sharing practices. The information can also enhance standardized guidance and evaluation activities to support recipients in submitting data to the tracking program.

## Figures and Tables

**Table 1:** Characteristics of 26 participating U.S. states *—Centers for Disease Control and Prevention (CDC) Public Health Tracking Program, 2019.

Category	Characteristic	No. (%)
Types of data **	Inpatient discharge	26 (100.0)
	Emergency department discharge	22 (84.6)
	Outpatient/non-inpatient discharge	8 (30.8)
	Observation stay files	8 (30.8)
	All-payer claims	6 (23.1)
Data provider	Hospital association	8 (30.8)
	Other health department, agency, commission, or board	18 (69.2)
	Sub-total	26 (100.0%)
Protected health information (PHI)	Record level identifiable data set with PHI	15 (57.7)
	Record level de-identified data set with PHI removed	7 (26.9)
	Aggregated data set (not record level)	2 (7.7)
	Other	2 (7.7)
	Sub-total	26 (100%)
Spatial resolution of data	Street address level	8 (30.8)
	Census tract level	3 (11.5)
	ZIP code level	9 (34.6)
	County level	1 (3.8)
	Other (block group, street, community, county, or town level)	5 (19.2)
	Sub-total	26 (100.0%)
Necessary elements to identify transfer	Yes, a combination of variables is provided	16 (61.5)
	Yes, patient ID is provided	6 (23.1)
	No, but data provide identifies/flags transfers	3 (11.5)
	No, data are too aggregated to identify transfers	1 (3.8)
	Sub-total	26 (100.0%)
Who is responsible for removing duplicates?	Data provider	12 (46.2)
	State program	9 (34.6)
	Other	5 (19.2)
	Sub-total	26 (100.0%)
Program conduct your own validation?	Yes	17 (65.4)
	No	9 (34.6)
	Sub-total	26 (100.0%)
How does your program correct errors/problems you find with the data (*n* = 17)	Our program asks the data agency/organization/department to correct and resubmit the data	9 (52.9)
	Our program corrects the error or corrects/notifies data steward	5(29.4)
	All the above	2 (11.8)
	Errors are not corrected	1 (5.9)
	Sub-total	17 (100.0%)
Any exclusion of data **	Veterans Affairs	23 (88.5)
	Tribal	20 (76.9)
	Federal facilities	21 (80.8)
	Specialty hospitals (e.g., psychiatric, cancer)	9 (34.6)
	Clinical access hospitals	3 (11.5)
	Other (e.g., prison, hospice, long-term, military hospitals)	8 (30.8)
	Sub-total	26 (100.0%)
Purposes of data use for environmental public health tracking **	To calculate nationally consistent data and measures (NCDMs) and send to CDC national tracking program	26 (100.0)
	To display non-NCDMs on our program’s state tracking portal	24 (92.3)
	To inform public health actions	24 (92.3)
	To conduct routine data analyses	23 (88.5)
	To create reports	18 (69.2)
	Other	6 (23.1)

* 25 states and New York City. ** Total for column is not 100% because of multiple choices.

**Table 2:** Exchange of health tracking data from bordering states—Centers for Disease Control and Prevention (CDC) Public Health Tracking Program, 2019.

Receiving Border Data?	State	Border States (Year of Data Received in 2019, Data Supplier **)
Yes, from all bordering states	Kansas	Missouri (2018, A), Colorado (2018, A), Oklahoma (2018, A)
	Michigan	Ohio (2018, A), Illinois/Indiana (2018, A), Wisconsin (2018, A)
	New Hampshire	Maine (2018, B), Massachusetts (2016, B), Vermont (2015, B)
Yes, from some but not all, bordering states	Minnesota	North Dakota (2017, A), Iowa (2017, A), South Dakota (2017, A)
	Missouri	Arkansas (2017, B), Illinois/Indiana (2017, B), Iowa (2017), Kansas (2017, B)
	New Mexico	Texas (2017, B)
	Vermont	New Hampshire (2015, A), Massachusetts (NP, A), New York (2016, A)
	Washington	Oregon (2016, O)
	Wisconsin	Minnesota (2018, B), Iowa (2018, B)

** A: Agency/organization that provides state data, B: Bordering state data agency/organization, O: Other, NP: Not provided.

## Supplementary Materials

The following are available online at https://www.mdpi.com/article/10.3390/ijerph18084356/s1, Table S1. Feedback from the Pilot.
