# Supplementary material for: Common and Unique Barriers to the Exchange of Administrative Healthcare Data in Environmental Public Health Tracking Program
Source: Int J Environ Res Public Health. 2021 Apr 20;18(8):4356. doi: 10.3390/ijerph18084356 (PMC8073470; doi:10.3390/ijerph18084356)
Supplement: Supplementary file 1 [file ijerph-18-04356-s001.zip › ijerph-1158414-supplementary.pdf]

Table S1. Feedback from the Pilot Survey.

Item	Questions	Feedback (# of Response)
Content	Are there questions that you feel are missing as we gather this information about your experience acquiring and using hospitalization data for Tracking?	None (3)
Logic	Issues with the survey logic	None (3)
Clarity	General edits to survey<br>Any trouble understanding a question—please describe	• Healthcare administrative claims data-> administrative healthcare data (1)<br>• Turned off progress bar (1)
Structure:	Have we left out any options (in the multi-choice and check box answers)	• For some questions, it may be preferable to include an "other" option if possible. Or an "I don't know."<br>• Maybe add a text box so the respondent can provide a brief explanation (2)<br>• There is no way to navigate backward (1)
Response time:	Time to complete the survey—you can include the time spent on information gathering if this is necessary	• 15–20 min (1)<br>• 40 min (1)<br>• One day (1)
Who from your program was needed to help complete the questions (e.g., PI, PM, hospitalization data lead)?		• No help needed (1)<br>• Need help from PI/PM, Hospitalization data lead (2)
